# Consumers’ Trade-Off between Nutrition and Health Claims under Regulation 1924/2006: Insights from a Choice Experiment Analysis

**DOI:** 10.3390/nu11122881

**Published:** 2019-11-26

**Authors:** Rosaria Viscecchia, Giuseppe Nocella, Biagia De Devitiis, Francesco Bimbo, Domenico Carlucci, Antonio Seccia, Gianluca Nardone

**Affiliations:** 1 Department of Science of Agriculture, Food and Environment University of Foggia, 71121 Foggia, Italy; biagia.dedevitiis@unifg.it (B.D.D.); francesco.bimbo@unifg.it (F.B.); gianluca.nardone@unifg.it (G.N.); 2 School of Agriculture, Policy and Development, University of Reading, Reading RG6 6BZ, UK; g.nocella@reading.ac.uk; 3 Department of Agricultural and Environmental Sciences, University of Bari “Aldo Moro”, 70126 Bari, Italy; domenico.carlucci@uniba.it; 4 Department of Humanities University of Foggia, 71121 Foggia, Italy; antonio.seccia@unifg.it

**Keywords:** nutrition claim, health claim, functional food, WTP, segmentation

## Abstract

The aim of this paper is to investigate consumers’ preferences and willingness to pay (WTP) for functional mozzarella cheese whose health benefits (reduced fat and enrichment in omega-3) are communicated by using nutrition claims (article 8) and health claims (articles 13 and 14) of the EU Regulation 1924/2006. In order to achieve the stated objective a choice survey was developed and administered to a sample of Italian respondents. The product attributes and attribute levels included in the choice experiment were obtained from in-depth interviews conducted with stakeholders working on the development of this new product in the Italian region of Puglia. Results show that many participants were not aware of functional food. Marketing segmentation performed via latent class analysis indicates that the development of this hypothetical product should be based on the addition of naturally enriched omega-3. In terms of health communication under Regulation 1924/2006, heterogeneity of preferences of the nine identified segments reveals that respondents have a clear preference for products from the Puglia region, for the combined nutrition claim over single nutrition claims and for the reduction of disease health claim (article 14) over the health claim (article 13). In monetary terms, willingness to pay for health claims is higher than nutrition claims.

## 1. Introduction

The “EU Regulation 1924/2006 on nutrition and health claims made on foods” makes a clear distinction between nutrition claims (article 8), health claims (article 13), and reduction of disease health claims (article 14). Nutrition claims inform consumers that a food has particular beneficial nutritional properties (e.g., either contains or does not contain a certain nutrient); permitted nutritional claims are listed in the Annex of Regulation 1924/2006. Health claims inform consumers that a relationship exists between a food or one of its constituents and health, while the reduction of disease health claims inform consumers that the consumption of a food or one of its constituents reduces a risk factor in the development of a human disease. To be included in the community list of permitted health claims, claims must be based on generally accepted scientific evidence and well understood by the average consumer [1,2,3].

The scientific substantiation of health claims (articles 13 and 14) is a challenging and long process because it requires a substantial effort in terms of financial and human resources on behalf of the food industry to get these claims approved by the European Food Safety Authority (EFSA). It has been estimated that in the European Union (EU), the costs of the scientific substantiation process of health claims range from €4.51 to €7.65 million, without taking into account clinical trials and other costs necessary to provide proprietary data to support applications to EFSA [4]. In order to use these claims manufacturers also face challenges related to changing lists of ingredients, wording of claims, and limited financial and research and development resources [5].

The list of scientific authorised health claims is continuously updated with regulation amendments (Table 1) based on assessments provided by the EFSA panel on dietary product, nutrition, and allergies. These evaluations have created uncertainty among food companies investing in research and development of new functional food products because of the high rate of rejections of submitted health claims [6,7]. Between July 2008 and March 2010 the European Commission (EC), after examining over 44,000 claims supplied by the Member States, submitted a list of 4.637 “general function” health claims to EFSA for scientific evaluation. However, at the end of June 2012 only 222 health claims (article 13) were authorised by EFSA [8]. As Table 1 shows, so far 243 health claims and 22 reduction of disease risk claims have been authorised under articles 13 and 14 of Regulation 1924/2006, respectively.

Manufacturers, using authorised food health claims, transfer information to consumers about positive effects that may be obtained from consuming their healthy products [9,10,11]. However, since the implementation of EU Regulation 1924/2006, it is not yet clear how information and beneficial effects communicated by nutrition and health claims can be perceived and understood by the average consumer [12].

A large body of literature explores how socio-demographic and economic characteristics of consumers influence their preferences towards nutrition and health claims. Even if some general patterns can be identified, the influence of socio-demographic and economic factors (gender, education, age, and income) remains undetermined [1,13,14,15,16,17,18,19,20,21,22,23]. 

Despite the fact that EU Regulation 1924/2006 was introduced in 2007, it appears that so far no studies have simultaneously compared consumers’ preferences and willingness to pay (WTP) for these three types of claims in the context of this regulation. Some studies explored WTP for different nutrition claims or compared consumers’ WTP for claims under article 8 with claims under articles 13 or 14. For example, Barreiro et al. (2010) [24] found a higher WTP for nutrition and health claims used on less healthy products (sausages) and that nutrition claims were not significant in the choice of the healthy product (yoghurt). Instead, Van Wezemael et al. (2014) [25] observed higher WTP for steaks conveying nutrition and health claims but also cross-cultural differences and heterogeneity of preferences both for types of nutrient (iron, fat, or protein) and claims (nutrition or health claim).

De Magistris and Lopez-Galan (2016) [27] found that consumers showed a positive willingness to pay for cheese with a reduced-fat claim, and a negative willingness to pay for cheese with a low salt claim. If both the claims “reduced-fat” and “low salt” were presented on the same package consumers were willing to pay a premium price. Lemken (2017) [28] also observed differences in consumers’ WTP for diverse types of nutrition claims conveyed on legumes pasta. He found that legume pasta advertised with nutrition claims showed a higher WTP than conventional pasta and in particular the fibre claim appeared to be superior to that of the protein claim. In a recent study, Lopez-Galan and de Magistris (2019) [29] found that emotional eating can have a negative impact in purchasing behaviour related to nutritional claims.

In light of this overview of past studies, this paper aims at evaluating how consumers’ preferences and WTP are influenced by the simultaneous presence of nutrition and health claims regulated by EU legislation 1924/2006. To achieve this objective, we explore the following research questions. To what extent, do nutrition and health claims under Regulation 1924/2006 influence consumer purchasing behaviour? Do consumers prefer nutrition, health, or reduction risk claims? Are consumers willing to pay more for claims regulated by article 8, 13, or 14? Do different nutrition and health claims generate heterogeneity of preferences? Is WTP of consumers who pay attention to claims the same as of those who do not attend information reported on food products? What socio-economic factors influence heterogeneity of preferences?

## 2. Materials and Methods

In order to achieve the stated objective a survey was developed on the basis of four in-depth interviews conducted with a food scientist, an animal husbandry expert, a dairy producer, and a marketing expert working on product development of this functional product. Experts stated that ongoing experiments were developing a functional mozzarella cheese whose consumption could help consumers to maintain normal levels of cholesterol because of an enhanced lipid composition obtained via reduced saturated fatty acids and/or enrichment of omega-3 (polyunsaturated fatty acids and conjugated linoleic acid). The enrichment of omega-3 was experimented either by adding this nutrient directly to milk or naturally feeding cows with flax seeds. Functional mozzarella cheese obtained in these two ways did not show any difference in terms of organoleptic characteristics with conventional mozzarella. Both methods did not impact very much on costs even if the natural enrichment of omega-3 was more expensive than when it was added directly to the milk. The only variation observed was linked to the production process that in comparison to conventional produce required a reduced temperature and spinning time of functional mozzarella cheese. Insights from in-depth interviews were fundamental to develop the questionnaire not only in relation to understanding what attributes and attribute levels this hypothetical functional mozzarella cheese might contain, but also to cherry pick the most appropriate nutritional and health claims for this new functional product from those approved under EU Regulation 1924/2006 (see Section 2.1).

The final questionnaire contained four sections. The first section collected information about respondents’ family history (heart attacks, strokes, and other pathologies) and concern for risk factors of cardiovascular diseases (e.g., high blood pressure, obesity, etc.), and knowledge of functional food products. The second section elicited information about participants’ consumption habits of mozzarella cheese. The third section presented the hypothetical market scenario for this product and the choice experiment. The last section collected information about socio-demographic and economic characteristics of respondents (gender, age, education, occupational status, income, etc.). The questionnaire was administered in 13 Italian cities (Bari, Bergamo, Bitritto, Cisterna di Latina, Fiumicino, Latina, Milano, Roma, Sanremo, Taranto, Treviso, Verona, Villorba) distributed in the four Italian Nielsen Areas. Data were collected between July and September 2013 by face-to-face interviews conducted by trained interviewers of an Italian marketing research company using the computer aided personal interview method. The sample was representative of the Italian population, stratified by age and sex according to demographic statistics provided by the national statistical institute.

### 2.1. The Choice Experiment and Econometric Analysis

The choice experiment was developed on the basis of information collected via in-depth interviews. As shown in Table 2, the attributes and relative levels used to develop the choice experiment design were: Origin (Not specified and made in Puglia), nutritional claims (No information, high in omega-3, reduced saturated fatty acids, high in omega-3, and reduced saturated fatty acids), health claims (No information, contributes to the maintenance of normal blood cholesterol levels, reduces cardiovascular disease risk), source of nutrient (No information, omega-3 added to the milk, and omega-3 present naturally in the milk), and price (+5%, and +10%, +20%, +40%). 

The choice experiment design was obtained by performing a *D-efficient* design using the NGene software [30]. The final design contained 36 choice sets blocked into groups of six, so we had six final versions of the survey instrument. Table 3 shows an example of the unlabelled choice set presented to respondents.

Consumers’ preferences for this hypothetical product were analysed by means of a discrete choice model [31,32]. The estimation of discrete choice models is based on Lancaster’s theory which postulates that consumers do not derive satisfaction from goods themselves but from their attributes and attribute levels. The Lancastrian approach assumes that each consumer chooses a single option yielding the greatest utility [33,34]. This class of econometric models has its basis in the random utility models, which represent the fundamental econometric approach to the analysis of consumer preferences within a discrete choice multi-attribute context [35]. Random utility models are based on the hypothesis that individuals make choices according to attributes of alternatives characterized by a degree of randomness [36]. According to Greene (2003) [37], random utility models for multiple choices indicate that for the *i*_th_ consumer faced with *J* alternatives, the utility of alternative *j* ($U_{ij}$) is

(1) $$U_{ij}=\;Z_{ij\;}^{Att}\beta_{ij}+\varepsilon_{ij}$$

where

- $Z_{ij\;}^{Att}\beta_{ij}$ is the deterministic component, i.e., a function of the sets of observable functional mozzarella attributes ($Z_{ij\;}^{Att}$) and a set of associated population parameters ($\beta_{ij}$) to be estimated;
- $\varepsilon_{ij}$ is the random component that captures variations in choices determined by omitted variables, measurement errors, and within- and between-individual variances.

The deterministic component was modelled as a function of functional mozzarella cheese products’ attributes assuming the Lancastrian approach that respondents can assign a subjective utility value to each product attribute, sum these values for each product to obtain a monotonic utility index, and select the product that gives them the highest utility. Thus, if a respondent makes her/his choice for a hypothetical functional mozzarella cheese product *j*, then, it is assumed that it is the maximum among the *J* utilities. The specification of this model depends on various conditions and assumptions about the distribution of the random component and on the way in which choice sets are developed and presented to respondents [37].

In this study, heterogeneity of preferences was estimated performing a latent class analysis using Latent Gold Choice 5.1 (Statistical Innovation Inc.). The number of classes was determined on the basis of statistical information criteria (Bayesian information criterion and the Consistent Akaike information criterion) [32]. Latent class analysis allows researchers to identify segments of individuals with identical preferences estimating the probability of membership to each class along with their respective class-specific preference weights. Thus, this segmentation technique is based on a likelihood model that permits statistical inference and is considered superior to traditional segmentation multivariate statistical techniques [34,38]. The following conditional logit model was estimated

(2) $$P\left(y_{ij}|Z_{ij}\right)=\sum_{x=1}^{k}P(x|Z_{i}^{Cov})\prod_{j=1}^{Ji}P(y_{it}|x,Z_{ij}^{Att})$$

where $P(y_{ij}|Z_{ij})$ is the probability of choosing alternative *j* conditional on the vectors of all covariate values of respondent *i*, *x* is the latent class that can take on values 1 ≤ *x* ≤ K, $Z_{i}^{Cov}$ refers to all characteristics of individual *i* and $Z_{ij}^{Att}$ is the vector of attribute values of respondent *i*. 

Finally, a growing number of studies recognises that respondents do not consider all attributes presented in the choice sets and that this kind of behaviour (non-attendance attribute) can lead to biased estimates of consumers’ preferences and willingness to pay [39,40,41,42,43]. In order to take account of this issue, we asked respondents to state what attributes they attended after having completed their choice tasks. Since the objective of this study is not to contrast modelling approaches that can account for stated or inferred attribute non-attendance, we only compare a latent class analysis where we include participants who stated to attend all attributes with a latent class analysis where participants who stated that they did not attend at least one attribute. Thus, for individuals who attended all attributes, the $Z_{i}^{Cov}$ of Equation (2) will only include the socio-demographic characteristics of respondents.

## 3. Results and Discussion

The final sample size for this study is 601 with an average age of participants of 46 (*s* = 14.8). Table 4 shows that the majority of participants were females (51.1%), with high school education (60.9%), more than two family members (56.2%), without children below the age of 14 (74.9%), from Northern Italy (50.0%), white collar (30.4%) and with a monthly net income less than €2000 (26.6%). During the survey many participants refused to disclose their income and thus for this variable 41.1% missing values were observed. For this reason, occupational status will be used as a proxy of income when estimating consumers’ preferences for functional mozzarella cheese.

### 3.1. Concern about Cardiovascular Disease and Knowledge of Functional Foods

About 38% of participants stated that in their families there were cases of heart attack, stroke, high blood pressure, or other illnesses such as angina pectoris, aneurism, etc. In addition, Table 5 shows to what extent respondents were worried about selected biological and lifestyle factors affecting cardiovascular diseases. The mean values of stress (4.52), cholesterol (4.21), blood pressure (4.12), and physical inactivity (4.08) indicate that participants are rather concerned about these risk factors. On the other hand, obesity, smoking, and diabetes with about one-quarter of respondents ‘not concerned at all’ and a mean of just above the mid-point of the proposed scale appear to be of less of a concern than previous risk factors. A test of reliability was performed (Cronbach’s Alpha = 0.929) to create an index of participants’ concern for cardiovascular diseases. The cardiovascular diseases concern index, obtained summing and averaging scores presented in Table 4, shows that on the average respondents ($\bar{X}$= 4.02; *s* = 1.64) were somewhat concerned about these risk factors. 

**Figure 1:**
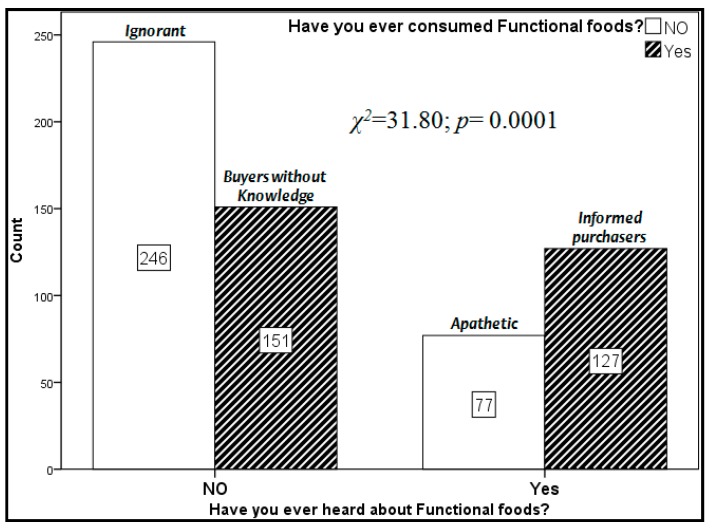
Relationship between knowledge and consumption of functional foods.

Furthermore, it was interesting to find out that only 66% of the participants had never heard the term functional foods before and that 53.7% had never consumed these products. The relationship between knowledge and consumption of functional foods was significant to the chi-square test (*χ*^2^ = 31.80; *p* = 0.0001). Cross tabulating the yes/no categories of these two variables in the following segments of consumers were identified: No knowledge/no consumption (unknowing), knowledge/no consumption (apathetic), buyers without knowledge, and informed purchasers. These four segments are illustrated in Figure 1 where it can be observed that the ignorant represent the largest segment (40.9%) of the sample, followed by buyers without knowledge (25.1%), apathetic (12.8%), and informed purchasers (21.1%). The identified segments are more or less consistent across Italian geographic areas with the ignorant category slightly bigger in the Middle in comparison to Northern and Southern Italy. There are more males than females in the unknowing and apathetic segments, while the opposite pattern was observed both for buyers without knowledge and informed purchasers. Age also affects these four segments with apathetic and informed buyers being on the average younger than ignorant and buyers without knowledge. Finally, the apathetic participants are more concerned about cardiovascular diseases than other segments with differences significant to the ANOVA one-way (*F* = 6.21; *df* = 3; *p* < 0.001).

### 3.2. Consumption Habits and Heterogeneity of Preferences for Nutritional and Health Claims

Mozzarella cheese or ‘Fiordilatte’ is a very popular Italian cheese which is consumed regularly by many consumers as confirmed by the results of this study where about 2/3 of respondents consume this product at least once a month. The average weekly quantity of mozzarella cheese purchased by respondents is 438 g (s = 406) with 43.4% of them spending weekly less than €5.00, 40.1% between €5.00 and €5.99, 12.5% between €10.00 and €14.99, and the remaining 4% more than €14.99. Mozzarella cheese is purchased exclusively in big retailers by about 57% of respondents, but 20% of them only buy this product in traditional shops and 4% only in local markets and specialized shops, while 19% of respondents buy mozzarella cheese both in traditional shops and big retailers. It is not surprising to observe that many respondents buy “Fiordilatte” in traditional and specialized shops because in these shops they can find unpackaged and very fresh mozzarella cheese that when consumed the day in which it is produced is a delicacy. In fact, 20.2% of respondents only buy the loose product, 41.4% only the packaged product, and 38.4% both loose and packaged mozzarella cheese.

The analysis of choice data show that 197 respondents stated to attend all attributes and 404 participants did not attend at least one attribute. Thus, in our sample a significant number of individuals did not process in full information provided in the choice sets. Taking into account the estimate of the 95% confidence interval for the proportion (*p* < 0.05), there should be between 63.5% and 71% individuals in the Italian population who ignore information for at least one attribute. This finding is confirmed also in other studies where the percentage of individuals who do not pay attention to at least one attribute seem to be more or less of the same magnitude 50% [39,40,41,42,43].

The development of functional mozzarella cheese designs and its marketing decisions depend on the selection of the right number of consumers’ segments that might buy this product. However, retaining the correct number of segments is a difficult task because experimental studies have shown that information criteria are influenced by sample sizes, parameter estimates, and model complexity [44,45]. Furthermore, the decision about the number of segments most appropriate for this data must also be informed by other factors, such as the pattern of significant parameters and relative signs can be informative [46]. Considering all these aspects and information criteria (LL, BIC, and CAIC) presented in Table 6, the structure of the data suggests the presence of up to four groups with different preferences for participants who stated attribute full attendance (SAFA) and five classes for those who stated non-attendance (SANA) for at least one of these attributes. In Appendix A, we also report the estimates of the full model in order to allow readers to make a comparison with the estimates of the SAFA and SANA models. 

Table 7 reports the parameter estimates for the attribute levels of the SAFA and SANA models, where ‘No information’ is the baseline for all discrete attributes of the choice experiment. The SAFA model shows four classes whose size ranges from 29% to 20% and according to the order of extraction have been named A (29%), B (26.9%), C (24.1%), and D (20%). The five classes of the SANA model show more variability in terms of size ranging from 29.8% to 14.4% and have been named E (29.8%), F (24.5%), G (15.9%), H (15.4%), I (14.4%). For both models, socio-demographic and economic characteristics were not significant other than the geographic area. The covariates of the SANA model also include the stated attribute non-attendance for origin, nutrition claims, health claims, source of omega-3, and price. 

With regards to parameters estimates of the SAFA model, the origin Puglia is significant in all four segments probably because SAFA respondents know that this region is renowned for mozzarella cheese production. The nutrition claim ‘Reduced fat’ is not significant in all classes, while price is negative and significant only in classes B and D. Moreover, the beta parameters of the other attributes of class A, are not significant or negative and significant (Omega-3 naturally present in the milk) and thus these participants were named ‘pro-tradition consumers’ because they are not attracted by functional mozzarella cheese. In class B, beta parameters are significant for the article 8 ‘Rich in omega-3’ and ‘Rich in Omega3 plus Reduced saturated fatty acids’, the article 14 ‘Helps to reduce cardio-vascular disease risk’ and for the attribute ‘Omega-3 added directly to milk’. These participants were named ‘pro-industry articles 8 and 14’ consumers because in addition to being interested in nutrition and health claims they show preferences for a production process where omega-3 is added directly to the milk. In class C, beta parameters are significant for the combined nutrition claim and for both health claims, but they are not interested in the way in which omega-3 is added to functional mozzarella. Participants belonging to this class were named ‘pro-EU Regulation 1924/20016’ consumers and they are likely to be from Southern Italy. In class D, beta parameters are significant for the combined articles 8 ‘Rich in Omega3 and Reduced saturated fatty acids’, the article 14 and for the attribute ‘Omega-3 already contained in the milk produced by cows’. These participants were named ‘pro-nature articles 8 and 14’ consumers because differently from those belonging to class B they are interested in a production process where omega-3 is naturally contained in milk because cows are fed with flax seeds. They are likely to be from Northern Italy.

As regards parameters estimates of the SANA model, origin is positive and significant in classes E, F, and H, negative and significant in class G, and not significant in class I. The nutrition claim ‘Reduced fat’ and the attribute ‘Omega-3 added to the milk’ are not significant in all classes, while price is negative and significant in all classes other than classes G. Class G appears to be a very distinct class of the SANA model because beta parameters are either not significant or negative and significant for the attributes ‘Puglia’ and the nutrition claim ‘Rich in omega-3’. Thus, participants belonging to this class were named ‘disinterested consumers’ as they preferred to have no information of this hypothetical product. The other classes of the SANA model appear to be more or less similar to classes of the SAFA model in relation to their preferences for nutrition and health claims. For example, respondents belonging to class E showed positive preferences for ‘omega-3 naturally present in the milk’ and for all types of claims and thus they were named ‘pro-nature EU regulation 1924/2006’. Participants in class F were only influenced positively by the combined nutrition claim and article 14 (pro-wellbeing article 8 and 14), and respondents in class I only by the combined nutrition claim and article 13 (pro-nature articles 8 and 13). Preferences of participants belonging to class H were only influenced by the Puglia provenance and thus they were named ‘pro-tradition’ as respondents of class A of the SAFA model. 

The covariates of attribute non-attendance are significant and negative for ‘price’ in Class E, for ‘source of Omega-3’ in class G, for ‘nutrition claims’ in class H, and for ‘origin in class I. This indicates that there is a high probability that respondents of these classes did not attend these attributes. On the other hand, ‘nutrition claims’ are positive and significant in class E and so ‘health claims’, ‘source of omega-3’, and ‘price’ in class I. In this case, the likelihood that participants attended these attributes is very high. Despite the fact that covariates results are difficult to interpret because several parameters are not significant, parameters that are significant can help us to evaluate the willingness to pay as presented in Table 8.

In monetary terms, Table 8 shows that, in both models, participants are willing to pay less when information is not provided, and that Puglia appears to be the most rewarding attribute. In regard to the SAFA model, intensity of preferences of respondents in class D is higher than those in class B for all attributes. For 100 g of functional mozzarella cheese, respondents in class D are willing to pay €0.07 more for Puglia, €0.14 for the combined nutrition claim, €0.20 for health claims, and €0.24 more omega-3 naturally enriched than participants in class B. Thus, combining the WTP of the various attributes, respondents in class B and class D might be willing to pay for this hypothetical product, respectively 2.20 and €8.40/Kg more than the product that they generally buy. In the SANA model, WTP estimates of class E are the largest than all classes identified in both models. Combining WTP of the various attributes, respondents of this class are willing to pay €11.60/Kg more than the product that they generally buy. Since respondents of this class did not attend the price it is likely that WTP estimates are biased. For class F and I WTP estimates of nutrition and health claims are a bit smaller than those observed in class B. Finally, the parameters estimates of the full model presented in the Appendix A show that for the seven classes identified results are similar to those of the SAFA and SANA models. The first three classes account for 67.2% of the make share. Origin appears to be the most attractive attribute and participants respond negatively to price increases other than for class E, which is price insensitive. Moreover, for the full model we observe a preference for the combination of nutrition claims and the article 14 claim. Instead, WTP estimates of the full model results are higher than those observed for the SAFA model. 

## 4. Conclusions

Even though large retailers, supermarkets, and shops market an enormous variety of functional food products, our results show only 21% of respondents were informed about functional food products. Most participants are uninformed about functional food products or buy these products without appropriate knowledge. Since functional food delivers health benefits that must be communicated with nutrition and health claims, manufactures should invest more in educational initiatives and marketing campaigns that aim at increasing an awareness of these products. By increasing consumers’ knowledge and awareness of functional foods and their health benefits, it is likely that consumers can pay more attention to nutrition and health claims. In regard to consumer preferences for functional mozzarella cheese, our study indicates that participants have a clear preference for Puglia, a well-known Italian region for the quality of this product. In relation to the investment on how to produce this product, only the ‘pro-industry articles 8 and 14’ participants showed a preference for omega-3 added directly to milk. On the other hand, omega-3 added naturally to milk is an attribute appealing both to ‘pro-nature articles 8 and 14’ participants of the SAFA model and to ‘pro-nature EU regulation 1924/2006’ and ‘pro-nature articles 8 and 13’ respondents of the SAFA model. Thus, stakeholders should invest more in developing breeding systems where cows are fed directly with flax seeds. In monetary terms this seems the most rewarding attribute because participants of these three classes are willing on the average to pay €1.80/Kg more than the product they usually buy.

In terms of choices about how to communicate health benefits of functional mozzarella cheese, participants’ preferences offer a clear indication in terms of article 8 of Regulation 1924/206. The single nutrition claim ‘reduced fat’ is not significant in all classes, while ‘Rich in omega-3’ is positive and significant in classes B and E, and negative and significant in classes C and G. Thus, the communication of health benefits obtained by adding nutrients (rich in omega-3) seems to attract consumers more than those gained by reducing nutrients (Reduced saturated fatty acids) for this specific product. Furthermore, the combined nutrition claim is positive and significant in six out of the nine classes identified in both models and on average participants are willing to pay about €1.00/Kg for communication delivered by the proposed combined claim. This result also corroborates a previous study [27] where consumers are WTP more for combined nutrition claims than a single nutrition claim reduced fat. In regard to health claims, results show that articles 13 and 14 were never negative and significant and thus it seems that in general, health claims influence consumers’ preferences more positively than nutrition claims. Comparing preferences between article 13 and article 14, the reduced risk health claim seems to be the most preferred being significant in five out of nine classes, while the former only in three classes. However, in monetary terms the average WTP for both health claims is about €1.30/Kg and thus a bit higher than the combined article 18 (€1.00/Kg). Moreover, in this case, our results confirm previous findings [24,47] in terms of preferences between article 13 and 14 of Regulation 1924/2006.

Finally, even if our WTP estimates appear to be acceptable in monetary terms, the high WTP observed for respondents belonging to class E of the SANA model confirms that attribute non-attendance can lead to biased results especially when participants do not attend price. In terms of health communication, marketers should pay more attention both to the Annex of EU Regulation 1924/2006 to see whether they can take advantage of combined nutrition claims and to the list of validated health claims because they seem to be more rewarding than nutrition claims. 

## Figures and Tables

**Table 1:** Amendments of EU Regulation 1924/2006 from 2007 to 2017.

Year of Amendment	Amendments of EU Regulation 1924/2006			
	Article 8 Nutrition Claims	Article 13 General Function (Health Claims)	Article 14.1a Reduction of Disease Risk (Health Claims)	Article14 1b Health Claims on Children’s Development and Health (Health Claims)
2007	Annex of nutrition claims			
2008	Regulation EC No 107/2008 and Regulation EC No 109/2008			
2009			Regulation 983/2009 (2 claims) Regulation 1024/2009 (1 claim)	Regulation 983/2009 (5 claims) Regulation 1024/2009 (1 claim)
2010	Regulation EC No 116/2010		Regulation 384/2010 (1 claim)	Regulation 957/2010 (2 claims)
2011	Regulation EC No 1169/2011			
2011			Regulation 665/2011 (2 claims) Regulation 1160/2011 (1 claim)	Regulation 440/2011 (1 claim)
2012	Regulation EC No (1047/2012)	Regulation EC No 432/2012 222 authorised health claims	Regulation 1048/2012 (1 claim)	
2013		3 Amendments Regulation EC No 432/2012 10 authorised health claims		
2014		1 Amendment Regulation EC No 432/2012 1 authorised health claims	Regulation 1135/2014 (1 claim) Regulation 1226/2014 (1 claim) Regulation 1228/2014 (3 claims)	
2015		3 Amendments Regulation EC No 432/2012 3 authorised health claims		
2016		3 Amendments Regulation EC No 432/2012 5 authorised health claims		
2017		2 Amendments Regulation EC No 432/2012 2 authorised health claims		.
Total of approved health claims		243	13	9

Source: Our Elaboration on EFSA data (www.efsa.europa.eu) [26]. EFSA (European Food Safety Authority).

**Table 2:** Attributes and attribute levels used in the choice experiment.

Attributes	Levels
Origin	Not specified; produced in Puglia
Nutritional claim	No information; high omega-3; reduced saturated fatty acids; high in omega-3, and reduced saturated fatty acids
Health claim	No information; contributes to the maintenance of normal blood cholesterol levels; helps to reduce risk of cardiovascular disease.
Information source of omega-3	No information, omega-3 added to the milk; omega-3 naturally present in the milk.
Price	+5%; +10%; +20%; +40%

**Table 3:** Example of a choice set.

**Please evaluate the attributes of these products and indicate your choice carefully. If none of these products satisfies your preferences indicate ‘none of these products’.**				
	**Product 1**	**Product 2**	**Product 3**	None of these products
Origin	Produced in Puglia	Not specified	Produced in Puglia	
Source of omega-3	No information	Omega-3 added to the milk	Omega-3 naturally present in the milk	
Nutritional claim	No information	High in omega-3 and reduced in saturated fatty acids	High in omega-3	
Health claim (Articles 13 and 14)	No information	Helps to reduce risk of cardiovascular diseases (Article 14).	Contributes to the maintenance of normal blood cholesterol levels (Article 13).	
Price	+ 5%	+ 40%	+ 20%	
Your choice	□	□	□	□

**Table 4:** Socio-demographic characteristics of respondents (*N* = 601).

Socio-Demographic and Economic Characteristics	Frequency	Percent
Gender (Female):	307	51.1
Education (High school or degree):		
Less than high school	108	19.6
High school	366	60.9
Degree	107	19.5
Family size (More than two members)	338	56.2
Presence of Children younger than 14 (Yes)	151	25.1
Occupational status:		
Unemployed	62	10.3
Blue collar	131	21.8
White collar	183	30.4
Manager	60	10.0
Pensioner	86	14.3
Housewife	79	13.1
Income:		
Under €2000	160	26.6
Between €2000 and €2999	116	19.3
Between €3000–€3999	38	6.3
€4000 or above	39	6.5
Missing values	248	41.3
Geographic area:		
North	301	50.0
Middle	150	25.0
South	150	25.0

**Table 5:** Concern for risk factors affecting cardiovascular diseases (*N* = 601).

	Concern	Not at All Concerned					Extremely Concerned		Mean(s)
Risk Factors		1	2	3	4	5	6	7	
Obesity		26.5%	11.8%	12.6%	12.1%	13.5%	9.5%	14.1%	3.59 (2.14)
Smoking		26.3%	8.8%	9.0%	11.3%	15.1%	12.1%	17.3%	3.86 (2.23)
Blood pressure		18.0%	8.2%	9.0%	18.3%	18.0%	12.6%	16.0%	4.12 (2.04)
Diabetes		23.0%	9.3%	12.5%	15.0%	15.0%	10.6%	14.6%	3.80 (2.10)
Cholesterol		11.6%	10.8%	12.1%	18.8%	17.5%	15.0%	14.1%	4.21 (1.90)
Triglycerides		14.6%	11.5%	11.6%	19.6%	18.0%	12.3%	12.3%	4.01 (1.92)
Stress		8.2%	5.0%	15.0%	20.1%	19.0%	16.3%	16.5%	4.52 (1.78)
Physical inactivity		13.8%	9.8%	11.1%	22.0%	18.1%	13.1%	12.0%	4.08 (1.88)

**Table 6:** Information criteria to select the number of classes for SAFA and SANA LC models *.

	SAFA LC Models				SANA LC Models			
Number of classes	Npar	LL	BIC	CAIC	Npar	LL	BIC	CAIC
1	10	−1289.44	2631.70	2641.70	10	−3190.41	6440.84	6450.84
2	23	−1190.75	2503.02	2526.02	28	−2848.49	5865.01	5893.01
3	36	−1153.23	2496.65	2532.65	46	−2671.54	5619.14	5665.14
4	49	−1116.25	2491.38	2540.38	64	−2560.59	5505.26	5569.26
5	62	−1086.41	2500.38	2562.38	82	−2490.60	5473.32	5555.32
6	75	−1067.68	2531.60	2606.60	100	−2439.32	5478.78	5578.78

* Npar = Number of estimated parameters.; LC = Latent class; LL = Log-likelihood; BIC (LL) = Bayesian information criterion based on the log-likelihood; CAIC (LL) = Consistent Akaike information criterion, based on the log-likelihood; SAFA = stated attribute full attendance; SANA = stated non-attendance.

**Table 7:** Comparison of maximum likelihood estimates between SAFA and SANA for consumer preferences towards functional mozzarella cheese.^1.^

	Classes	SAFA (*N* = 197)				SANA (*N* = 404)				
Attributes		*β*_A (29%)_	*β*_B (26.9%)_	*β*_C (24.1%)_	*β*_D (20%)_	*β*_E (29.8%)_	*β*_F (24.5%)_	*β*_G (15.9%)_	*β*_H (15.4%)_	*β*_I (14.4%)_
**Origin:**		1.66 (6.17)***	1.07 (7.45)***	0.36 (3.27)**	0.47 (2.92)**	0.90 (8.46)***	1.72 (7.71)***	−0.34 (3.01)**	0.67 (3.94)***	−0.05 (0.47)
Puglia										
**Nutrition claims (Article 8):**										
Reduced fat		0.03 (0.12)	−0.041 (1.19)	0.32 (1.06)	−0.26 (0.59)	0.16 (0.79.)	−0.18 (0.74)	0.11 (0.47)	−0.68 (1.57)	−0.69 (1.17)
Rich in omega-3		0.21 (1.27)	0.49 (2.31)*	−0.65 (2.74)**	0.42 (1.68)	0.32 (2.68)	0.11 (0.62)	−0.48 (2.48)	−0.44 (1.36)	0.33 (1.07)
Rich in omega-3 and reduced fat		0.44 (1.82)	0.48 (2.39)**	1.27 (6.40)***	0.52 (2.39)**	0.45 (3.68)***	0.60 (2.89)**	−0.17 (1.11)	0.39 (1.82)	0.96 (3.92)***
**Health claims (Articles 13 and 14):**										
Contributes to the maintenance of normal blood cholesterol levels (Article 13).		0.18 (0.96)	0.18 (0.87)	0.75 (4.07)***	−0.09 (0.47)	0.26 (2.38)**	0.25. (1.45)	−0.28 (1.33)	−0.16 (0.77)	0.39 (2.11)**
Helps to reduce cardiovascular diseases risk (Article 14).		−0.20 (1.02)	0.43 (2.15)**	0.47 (3.11)**	0.65 (3.49)***	0.44 (4.09)***	0.47 (2.36)**	−0.08 (0.51)	−0.03 (0.13)	0.24 (1.38)
**Information Source of omega-3:**										
Omega-3 added to the milk		0.27 (1.75)	0.35 (2.22)**	0.22 (1.37)	−0.10 (0.64)	−0.03 (0.35)	0.18 (1.24)	0.11 (0.57)	0.32 (1.64)	0.23 (1.54)
Omega-3 naturally present in the milk		−0.60 (2.99)**	0.35 (1.47)	0.12 (0.62)	0.65 (2.98)**	0.58 (4.30)***	−0.15 (0.71)	−0.31 (0.87)	−0.04 (0.15)	0.46 (2.07)**
**Price**		0.36 (0.71)	−10.35 (7.22)***	0.10 (0.23)	−2.74 (4.98)***	−2.05 (5.31)***	−13.49 (8.77)***	0.51 (1.04)	−24.19 (5.71)***	−9.93 (6.46)***
**No buy**		−1.08 (2.61)**	−1.11 (5.70)***	−4.17 (0.97)	−1.27 (5.46)***	−1.23 (8.57)	−1.57 (7.27)***	−0.16 (0.98)	−0.51 (2.12)**	−1.65 (7.02)***
**Intercept**		1.00 (2.21)**	0.53 (1.10)	−0.60 (0.65)	−0.92 (0.96)	1.08 (2.22)**	−1.24 (0.81)	0.39 (0.61)	0.34 (0.72)	−0.56 (0.98)
**Covariates**										
**Italian geographic areas:**										
Northern Italy		−0.06 (0.09)	0.83 (1.19)	−3.30 (1.87)	2.54 (2.35)**	−0.02 (0.10)	−0.62 (2.47)	0.75 (1.72)	0.14 (0.64)	−0.25 (0.85)
Central Italy		−0.33 (0.70)	0.14 (0.28)	0.51 (0.54)	−0.32 (0.31)	−0.18 (0.61)	−0.09 (0.41)	0.27 (0.57)	0.04 (0.14)	−0.04 (0.09)
Southern Italy		0.39 (0.57)	−0.97 (1.25)	2.79 (2.64)**	−2.22 (1.25)	0.20 (0.61)	0.72 (2.28)**	−1.03 (1.23)	−0.18 (0.54)	0.29 (0.75)
**Stated attribute non-attendance:**										
Origin		-	-	-	-	0.39 (0.88)	2.24 (1.51)	−0.63 (1.53)	−0.55 (1.33)	−1.44 (3.36)***
Nutrition claims		-	-	-	-	0.48 (2.58)**	0.15 (0.95)	−0.20 (1.04)	−0.67 (3.20)***	0.23 (1.00)
Health claims		-	-	-	-	−0.17 (1.00)	−0.11 (0.65)	−0.34 (1.90)	−0.07 (0.43)	0.70 (2.59)**
Source of omega-3		-	-	-	-	0.29 (1.76)	−0.09 (0.54)	−0.50 (2.41)**	−0.29 (1.78)	0.59 (2.44)**
Price		-	-	-	-	−0.97 (4.97)***	0.30 (1.09)	−0.44 (1.85)	-0.02 (0.08)	1.12 (2.82)**

^1^ In brackets z values: *** Significant at 0.001; ** Significant at 0.01; * Significant at 0.05.

**Table 8:** Comparison of WTP (willingness to pay) for mozzarella functional cheese attributes between SAFA and SANA models*.

	Classes	SAFA WTP (βattribute/βprice)		SANA WTP (βattribute/βprice)			
Attribute Levels		Class B	Class D	Class E	Class F	Class H	Class I
Puglia		0.10	0.17	0.44	0.13	0.03	NS
No information		−0.05	−0.25	−0.46	−0.04	0.03	−0.02
Reduced fat		NS	NS	NS	NS	NS	NS
Rich in omega-3		0.05	NS	0.16	NS	NS	NS
Reduced fat+Rich in Omega-3		0.05	0.19	0.22	0.04	NS	0.04
No information		NS	NS	−0.34	−0.05	NS	−0.03
Article 13		0.04	0.24	0.12	NS	NS	0.02
Article 14		0.04	0.24	0.22	0.03	NS	NS
No information		NS	NS	−0.27	NS	NS	−0.03
Added directly to milk		0.03	NS	NS	NS	NS	NS
Naturally enriched		NS	0.24	0.28	NS	NS	0.02

* Values in EURO; NS = Not significant.

## Appendix A

**Table A1:** Information criteria to select the number of classes for SAFA and SANA LC models *.

	LC Models of the Full Sample			
Number of Classes	Npar	LL	BIC	CAIC
1	10	−4569.83	9203.64	9213.64
2	28	−4126.16	8431.49	8459.49
3	46	−3917.62	8129.58	8175.58
4	64	−3780.12	7969.75	8033.75
5	82	−3692.52	7909.72	7991.72
6	100	−3619.36	7878.59	7978.59
7	118	−3560.7246	7876.48	7994.48
8	136	−3514.1645	7898.54	8034.54

* Npar = Number of estimated parameters.; LL = Log-likelihood; BIC (LL) = Bayesian information criterion based on the log-likelihood; CAIC (LL) = Consistent Akaike information criterion, based on the log-likelihood.

**Table A2:** Maximum likelihood estimates of the full sample for consumer preferences towards functional mozzarella cheese.^1.^

	Classes	Full sample						
Attributes		*β*_A (27.8%)_	*β*_B (24.3 %)_	*β*_C (15.1 %)_	*β*_D (12%)_	*β*_E (9.2%)_	*β*_F (7.5%)_	*β*_G (4.1%)_
**Origin:**		1.30 (10.83)*****	1.35 (13.91)*****	−0.15 (1.77)	0.47 (4.53)****	−0.21 (1.77)	1.25 (5.22)****	0.22 (0.96)
Puglia								
**Nutrition claims (Article 8):**								
Reduced fat		0.02 (0.13)	−0.17 (0.88)	−0.14 (0.55)	0.30 (1.14)	0.18 (1.75)	−0.74 (1.31)	−2.69 (0.39)
Rich in omega-3		0.24 (2.38) ****	0.23 (1.73.)	0.20 (1.20)	−0.35. (1.52)	−0.59 (2.62)****	−0.82. (1.71)	1.82 (0.77)
Rich in omega-3 and reduced fat		0.36 (2.96) ****	0.54 (4.42) *****	0.23 (1.74)	1.20 (6.23)*****	0.10 (0.50)	0.28 (0.92)	1.95 (0.84)
**Health claims (Articles 13 & 14):**								
Contributes to the maintenance of normal blood cholesterol levels (Article 13).		0.09 (1.01)	0.28 (2.10)	0.05 (0.46)	0.69 (3.72)*****	−0.37 (2.40)****	0.04 (0.16)	−0.53 (1.30)
Helps to reduce cardiovascular diseases risk (Article 14).		0.15 (1.41)	0.40 (3.01)*****	0.32 (2.75 ****	0.52 (3.88)*****	−0.04 (0.22)	0.15 (0.46)	0.73 (1.74.)
**Information Source of omega-3:**								
Omega-3 added to the milk		0.06 (0.69)	0.26 (2.58)****	0.03 (0.33)	0.18 (1.37)	0.28 (1.67)	0.59 (1.91)	0.18 (0.53)
Omega-3 naturally present in the milk		0.04 (0.34)	−0.18 (1.24)	0.54 (3.68)*****	0.27 (1.51)	−0.47 (2.33)****	−0.05 (0.11)	0.66 (1.48)
**Price**		−0.44 (3.14) *****	−13.28. (12.48)*****	−2.22 (5.20)*****	−0.10 (0.23)	0.94 (2.54)****	−51.97 (6.29)*****	−40.21 (5.27)*****
**No buy**		−0.85 (5.88) *****	−1.79 (11.29)*****	−1.66 (7.95)*****	−4.28 (1.15)	0.48 (4.16)*****	−1.78 (4.62)*****	−2.19 (1.75)
**Intercept**		1.67 (2.16)****	1.21 (2.60)****	2.03 (2.97)****	5.14 (1.67)	0.66 (0.86)	1.15. (1.60)	−1.57 (0.78)
**Covariates**								
**Italian geographic areas:**								
Northern Italy		0.06. (0.28)	−0.21 (1.03)	0.89 (3.34)*****	2.06 (3.03)*****	1.37 (3.86)*****	0.17 (0.65)	−0.23 (0.52)
Central Italy		−0.30 (1.41)	−0.04 (0.22)	0.06 (0.23)	−0.16 (0.39.)	0.21 (0.52)	0.12 (0.46)	0.10 (0.22)
Southern Italy		0.24 (0.98)	1.12 (0.96)	−0.06 (2.40)****	2.21 (5.21)*****	−1.59 (2.68)****	-0.29 (0.31)	0.13 (0.27)
**Stated attribute non-attendance:**								
Origin		1.47 (3.07)*****	0.85 (2.91)****	−0.79 (3.60)*****	1.13 (1.88)	−0.49 (1.98)***	0.08 (0.27)	−2.25 (5.06.)
Nutrition claims		0.19 (0.59)	−0.18 (0.56)	0.02 (0.05)	1.95 (1.13)	−0.81 (2.29)****	−0.76 (2.16)****	−0.40 (0.92)
Health claims		−0.29 (0.87)	−0.30 (0.93)	−0.28 (0.85)	2.56 (1.47)	−0.99 (1.13)	−0.43 (1.22)	−0.88 (2.11)***
Source of omega-3		−0.15 (0.49)	−0.44 (1.51)	−0.35 (1.06)	1.63 (1.00)	−0.62 (1.86)	−0.83 (2.11)****	0.76 (1.01)
Price		−1.04 (2.94)****	0.68 (1.18)	−0.37 (0.96)	−0.92 (2.19)****	0.10 (0.20)	−0.61 (0.49)	1.85 (0.96)

^1^ In brackets z values: *** Significant at 0.001; ** Significant at 0.01; * Significant at 0.05.

**Table A3:** WTP for mozzarella functional cheese attributes of the full sample (*N* = 601)*.

	WTP Full Sample (βattribute/βprice)			
Attribute Levels	Class A	Class B	Class C	Class F
Puglia	2.95	1.35		1.25
No information	−1.41	−0.05	NS	0.02
Reduced fat	NS	NS	NS	NS
Rich in omega-3	0.55	NS	NS	NS
RF and RO-3	0.82	0.04	NS	NS
No information	−0.25	−0.05	−0.13	NS
Article 13	NS	0.02	NS	NS
Article 14	NS	0.03	0.11	NS
No information	NS	NS	−0.20	NS
Added directly to milk	NS	0.02	NS	NS
Naturally enriched	NS	NS	0.18	NS

* Values in EURO; NS = Not significant.
